# Supplementary material for: Insight into the Speciation of Heavy Metals in the Contaminated Soil Incubated with Corn Cob-Derived Biochar and Apatite
Source: Molecules. 2023 Feb 27;28(5):2225. doi: 10.3390/molecules28052225 (PMC10005082; doi:10.3390/molecules28052225)
Supplement: Supplementary file 1 [file molecules-28-02225-s001.zip › molecules-2182589-supplementary.pdf]

Supplementary Information

Table S1. The proportion of chemical fractions in soils after 30 days of incubation with biochar and apatite ore (%).

Metal	Sample	F1 (%)	F2 (%)	F3 (%)	F4 (%)	F5 (%)
**Pb**	CS	10.4	67.3	8.5	0.6	13.2
	CB4:3	11.6	63.2	9.5	0.7	14.9
	CB4:5	9.1	67.2	8.9	1.0	13.8
	CB4:10	8.0	65.9	8.9	1.6	15.6
	CB4A3	10.6	67.1	8.6	0.6	13.1
	CB4A5	8.6	62.0	8.6	0.7	20.1
	CB6:3	7.8	68.4	9.3	0.9	13.6
	CB6:5	9.8	64.2	10.0	0.7	15.2
	CB6:10	7.9	64.3	10.1	0.7	16.9
	CB6A3	9.9	65.2	9.6	0.8	14.5
	CB6A5	9.5	62.3	10.5	0.9	16.9
**Zn**	CS	16.4	28.0	28.3	0.4	26.9
	CB4:3	14.3	29.7	25.8	0.9	29.3
	CB4:5	14.5	32.0	22.8	0.8	29.9
	CB4:10	12.1	30.9	21.9	2.4	32.7
	CB4A3	13.3	30.7	22.7	2.1	31.2
	CB4A5	11.4	30.5	22.0	2.4	33.7
	CB6:3	15.5	30.4	23.2	0.9	30.0
	CB6:5	13.5	33.1	26.2	0.9	26.3
	CB6:10	13.1	30.0	27.3	0.8	28.9
	CB6A3	15.7	34.2	25.8	0.7	23.6
	CB6A5	14.3	28.8	26.6	0.8	29.4

Table S2.: Operating parameter of microwave digestion system for digesting soil samples

Nr	Operating parameters of microwave digestion Mars 6	
1	Power	(~ 1200 W)
2	Rising temperature duration	10 minutes
3	Holding time	15 minutes
4	Digestion temperature	170 °C
5	Cooling time	20 minutes

Table S3. Parameter of ICP-MS Agilent 7900 and recovery values of elements in sediment reference material MESS-4

Operating conditions of ICP-MS Agilent 7900			
High-Frequency Power (w)	~1600		
Sampling depth (mm)	~10		
Carrier gas flow rate (L/min)	~0,7		
Auxiliary gas flow rate (L/min)	~0,3		
Peristaltic nebulizer (concentric glass)	MicroMist		
Spray chamber temperature (°C)	2		
Helium gas flow rate (mL/min)	~4,3		
Hydrogen gas flow rate (mL/min)	~4,2		
Peristaltic pump speed	0,1 (0,5 mL/min)		
Internal standard	^{115}In		
Isotopes	^{208}Pb, ^{111}Cd, ^{66}Zn		
LOD and LOQ values of metals using ICP-MS	Element	LOD (ng/L)	LOQ (ng/L)
	Pb	13.7	41.5
	Cd	2.4	7.2
	Zn	1.5	4.5
Recovery values of metal elements (%) for MESS-4	Element	Recovery (%)	RSD (%)
	Pb	109.27	9.82
	Cd	92.11	15.88
	Zn	103.22	13.47

Table S4. Tessier's sequential extraction procedure

Step	Fractions	Reagent extractions/ conditions
1	Exchangeable (F1)	10 mL NH_4OAc 1M, 1 hour shaking
2	Carbonates bound (F2)	20 mL NH_4OAc 1M/HOAc pH = 5, 5 hours shaking
3	Fe and Mn oxides bound (F3)	20 mL $NH_2OH.HCl$ 0,04M/HOAc 25%, 5 hours shaking
4	Organic matter bound (F4)	10 mL NH_4OAc 3,2M + HNO_3 20%, 0.5 hour shaking
5	Residual (F5)	50 mL HNO_3 + HCl (v/v = 1:3), 0.5 hour shaking
